# The FcεRI signaling pathway is involved in the pathogenesis of lacrimal gland benign lymphoepithelial lesions as shown by transcriptomic analysis

**DOI:** 10.1038/s41598-021-01395-z

**Published:** 2021-11-08

**Authors:** Jing Li, Rui Liu, Mei Sun, Jinjin Wang, Nan Wang, Xuan Zhang, Xin Ge, Jianmin Ma

**Affiliations:** Beijing Institute of Ophthalmology, Beijing Tongren Eye Center, Beijing Tongren Hospital, Capital Medical University, Beijing Ophthalmology & Visual Sciences Key Laboratory, Beijing, 100730 China

**Keywords:** Immunology, Diseases, Pathogenesis

## Abstract

This study aimed to analyze the role of the FcepsilonRI (FcεRI) signaling pathway in the pathogenesis of benign lymphoepithelial lesion of lacrimal gland (LGBLEL). Transcriptomic analysis was performed on LGBLEL and orbital cavernous hemangioma (CH) patients diagnosed via histopathology in Beijing Tongren Hospital, Capital Medical University, between July 2010 and October 2013. Four LGBLEL and three orbital CH patients, diagnosed between October 2018 and August 2019, were randomly selected as experimental and control groups, respectively. RT-PCR, immunohistochemical staining, and western blotting were used to verify genes and proteins related to the FcεRI signaling pathway. Transcriptomic analysis showed that the FcεRI signaling pathway was upregulated in the LGBLEL compared with the CH group. The mRNA expression levels of important genes including SYK, p38, JNK, PI3K, and ERK were significantly increased in the LGBLEL group (P = 0.0066, P = 0.0002, P = 0.0003, P < 0.0001, P < 0.0001, respectively). Immunohistochemical staining results showed that SYK, p38, and ERK were positively expressed in LGBLEL, while JNK and PI3K were not. The protein contents of P-SYK, P-p38, P-JNK, P-PI3K, and P-ERK were significantly higher in the LGBLEL than in the CH group (P = 0.0169, P = 0.0074, P = 0.0046, P = 0.0157, P = 0.0156, respectively). The FcεRI signaling pathway participates in the pathogenesis of LGBLEL.

## Introduction

Lacrimal gland benign lymphoepithelial lesion (LGBLEL) is an inflammatory disease common in middle-aged women. Symptoms of LGBLEL include swelling of the eyelids and diffuse enlargement of the lacrimal glands. The typical pathological manifestations are diffuse infiltration of lymphocytes and plasma cells in lacrimal gland tissue, atrophy and disappearance of glands, and hyperplasia of fibrous tissue^1^. It is now recognized that lymphoepithelial lesions in glands can result from a variety of disorders ranging from reactive lymphoid proliferations to overt B cell lymphomas of mucosal associated lymphoid tissue^2,3^. Previous studies have suggested that BLEL is part of Sjögren's syndrome (SS) due to the similar pathologic features^4^; however, other systemic diseases and symptoms, such as salivary gland enlargement, dry eye, and dry mouth, are less commonly associated with LGBLEL than they are with SS. Therefore, the term LGBLEL has been used to describe benign lymphoepithelial changes in lacrimal glands associated with unilateral or bilateral gland enlargement in the absence of the clinical signs of SS (dry eyes and mouth)^3–5^.

At present, the pathogenesis and mechanisms involved in LGBLEL are not clear, though several different hypotheses suggest that LGBLEL may be related to sex hormones, basal cell infiltration, autoimmune disease, and/or correlated with IgG4 levels^1,6,7^. Increased expression of IgG4 in serum and tissue has been found in some cases of LGBLEL. Therefore, LGBLEL with positive expression of IgG4 is considered IgG4-related ocular disease (IgG4-ROD)^8^. IgG4-ROD is an immune-associated inflammatory disease, with etiology that may be related to SS, allergic rhinitis, dry eye, and asthma^9,10^.

Studies have found that complement mediated signaling pathways, T cell signaling pathways, and B cell signaling pathways are all involved in the pathogenesis of LGBLEL^1,11,12^. FcεRI is a high-affinity IgE receptor, which has been shown to be involved in type I hypersensitivities, such as allergic asthma^13^. Activation of the classical IgE-mediated FcεRI signaling pathway can directly lead to increased secretion of cytokines, such as interleukin (IL)-3, IL-4, IL-5, IL-13, and tumor necrosis factor-α, leading to activation of helper T2 cells (Th2) and eosinophils, as well as aggravation of the inflammatory response, which is manifested by increased concentrations of serum IgG4 and IgE. IgE has a positive feedback effect on the FcεRI signaling pathway. Non-IgE-mediated allergic reactions can be caused by activation of IgG immune-related complexes and complement systems^14–16^. Activation of the FcεRI signaling pathway can lead to the release of inflammatory mediators and promote a chronic inflammatory response. Continued inflammation can lead to long-term lacrimal tissue injury, fibrosis, remodeling, and even malignant transformation. Therefore, we hypothesize that the IgE-mediated FcεRI signaling pathway may be involved in the pathogenesis of LGBLEL.

In this study, transcriptomics analysis was used to analyze the role of the FcεRI signaling pathway in the pathogenesis of LGBLEL. Western blotting, real time-PCR (RT-PCR), immunohistochemical staining were used to verify the expression of genes and proteins related to the FcεRI signaling pathway in LGBLEL tissues and to study the mechanism of action of the FcεRI signaling pathway.

## Subjects and methods

### Subjects

**Figure 1:**
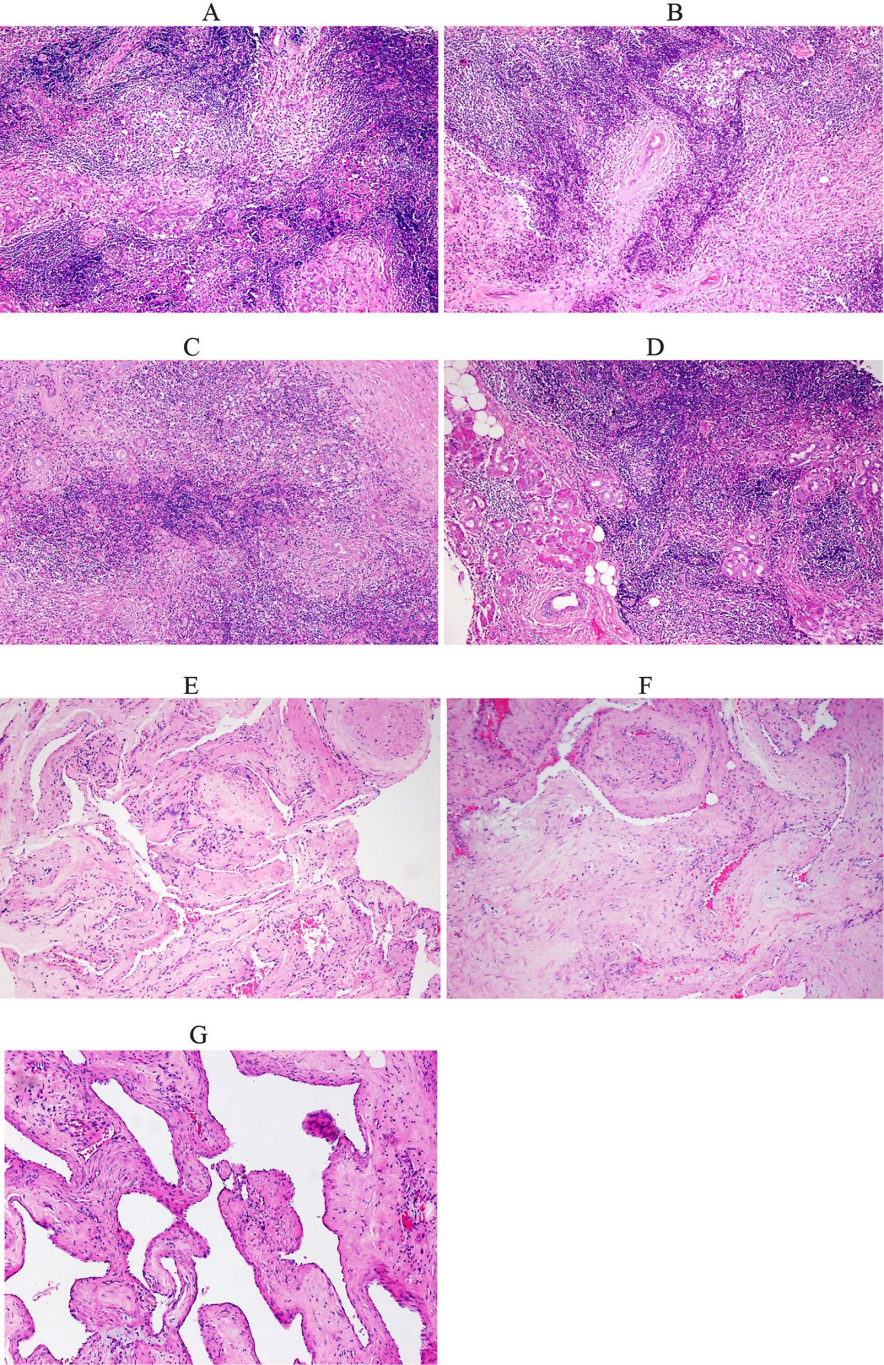
Pathological manifestations of benign lymphoepithelial lesions of lacrimal gland and cavernous hemangioma. (**A**–**D**) Diffuse infiltration of lymphocytes and plasma cells, atrophy and disappearance of glands, and hyperplasia of fibrous tissue. (**E**–**G**) The blood filled spaces are relatively uniform in size and are separated by fibrous tissue. (Hematoxylin–Eosin Staining, magnification × 100).

This study included an experimental group comprised of LGBLEL patients and a control group comprised of cavernous hemangioma (CH) patients. The diagnosis of LGBLEL and CH was mainly based on histopathological examination, as well as the typical pathological manifestations of LGBLEL, including diffuse infiltration of lymphocytes and plasma cells in the lacrimal gland tissue, atrophy and disappearance of glands, and hyperplasia of fibrous tissue (Fig. 1A–D). CH was composed of plexiform, thin-walled vascular sinus-like structures, separated by nerve fibers (Fig. 1E–G). Nine LGBLEL and nine CH patients, diagnosed via histopathological examination by two experienced pathologists in Beijing Tongren Hospital, Capital Medical University, between August 2010 and March 2013, were randomly selected for transcriptome sequencing. Four cases of LGBLEL and three cases of CH, diagnosed between October 2018 and August 2019, were randomly selected as the experimental and control groups, respectively, for a verification experiment. All the samples were selected at random, and there were no statistically significant differences in age or sex among groups (Table 1).

**Table 1:** Characteristics of patients with lacrimal gland benign lymphoepithelial lesion and orbital cavernous hemangioma.

Sample	Transcriptomics		Verification experiment	
	Male/female	Median age (years)	Male/female	Median age (years)
LGBLEL	1/8	43	1/3	46
CH	2/7	52	2/1	48
P value	1.000*	0.504^#^	0.486*	0.657^#^

*Fisher’s exact probability method.

^#^Wilcoxon-Mann–Whitney U two sample test.

Informed consent was obtained for experiments involving human participants (including the use of tissue samples). This study was conducted in accordance with the principles of the Helsinki Declaration and was approved by the Ethics Committee of Beijing Tongren Hospital, Capital Medical University (TRECKY2019-093).

### Tissue and blood specimen collection

Pathological tissue samples from patients with LGBLEL and orbital CH were collected by clinicians during surgery and transferred to a standardized laboratory for study. Portions of the tissue specimens were stored in a freezer at − 80 °C for later use; the other parts were soaked in 10% formalin for paraffin embedding and sectioning.

### Transcriptomic analysis

Total RNA was extracted via a mirVana miRNA Isolation Kit (Ambion, Texas, USA), according to the manufacturer’s instructions and RNA quality was evaluated using an Agilent 2100 Bioanalyzer (Agilent, California, USA). The dataset was sequenced on an Illumina HiSeq 2000 (100-bp paired-end), and quality control processes were performed using the FASTQ software (http://hannonlab.cshl.edu/fastx_toolkit/, version0.0.13)^17^. The Benjamini–Hochberg procedure for multiple testing correction was used to control the false discovery rate (FDR) and significant differentially expressed genes (DEGs) were selected with |log2FC|> 1 and FDR < 0.01. Gene ontology (GO) functional annotation for biological processes and the Kyoto Encyclopedia of Genes and Genomes (KEGG) pathways enrichment to determine differentially expressed genes (DEGs) between LGBLEL and CH were performed using the Database for Annotation, Visualization and Integrated Discovery (DAVID) online platform, version 6.8 (https://david.ncifcrf.gov/). Significantly enriched GO terms and KEGG pathways with an FDR < 0.05 were selected.

### Reverse transcription polymerase chain reaction (RT-PCR)

The pathological tissue was cut, lysate added, and RNA extracted with TRIzol reagent (Invitrogen) according to the manufacturer's instructions. PCR primers were designed, and the cDNA of total RNA was synthesized via reverse transcription according to RT kit instructions and was used as a template for RT-PCR (Eppendorf, 533353658)^18^. The results were analyzed using the CopyCaller® (version 2.1; Applied Biosystems, USA) software. The PCR reaction conditions were 95 °C/3 min, 95 °C/30 s, 55 °C/20 s, 72 °C/20 s, and 40 cycles; GAPDH was used as the internal reference. Primer sequence information is shown in Table 2.

**Table 2:** Sequences of real-time PCR primers.

Gene	Primer Sequence 5′–3′
GAPDH	F: GCCTTCCGTGTCCCCACTGC
	R: GGCTGGTGGTCCAGGGGTCT
SYK	F: TGAAGCAGACATGGAACCTG
	R: CAATTTGCTCAGATTCTTCCC
p38	F: GGTTACGTGTGGCAGTGAAG
	R: CAATGTTGTTCAGATCTGCCC
ERK	F: TGTTGCAGATCCAGACCATG
	R: AGGTCTTCTTGTGATGGGGA
JNK	F: TCAGAATCAGACTCATGCCA
	R: CATCTGAATCACTTGGCAAAG
PI3K	F: CAACAACTGCATCTTCATCG
	R: TCTCTTCTCCGTTCTTGAGG

### Immunohistochemical staining

The diseased tissue sections were dewaxed, incubated at room temperature for 5–10 min, washed with distilled water, and soaked in PBS for 5 min. Drops of primary antibody were added, and the tissue sections incubated overnight at 4 °C. The tissue was then washed three times with PBS, biotin-labeled secondary antibody was added after 5 min, and the tissue sections were incubated at 37 °C for 30 min^19^. The tissue was again washed three times with PBS, DAB stained, rinsed with water, hematoxylin stained, mounted, and imaged under a microscope (Olympus CX41, Tokyo, Japan). Polyclonal Rabbit IgG (ab37415, Abcam) was selected for isotype control antibody, which immunohistochemically showed all negative in 4 LGBLEL patients. The primary antibodies used for the immunohistochemical staining of proteins related to FcεRI signaling pathway were spleen tyrosine kinase (SYK) (AF3314; Affinity Biosciences, Beijing, China), p38 (mitogen-activated protein kinase, MAPK) (AF4001; Affinity Biosciences), c-Jun N-terminal kinase (JNK) (AF3318; Affinity Biosciences), phosphoinositide 3-kinase (PI3K) (AF3241; Affinity Biosciences) and extracellular signal-regulated kinases (ERK) (AF1015; Affinity Biosciences).

### Western blotting

The diseased tissue was cut into pieces, lysate added, centrifuged, and the protein concentration detected using a BCA protein assay kit (Beyotine, Wuhan, China). An electrophoretic gel was prepared and the protein samples were mixed with 5 × loading buffer and treated at 100 °C for 5 min. An amount of 100 μg of cell protein was collected from each well, and electrophoresis was performed with a constant current power source of 15 mA per gel. The transfer tank was filled with electro-transfer solution to start the film transfer and the color-developing reagent system was added to the protein membrane^20^. The ratio of the target to GAPDH light density was regarded as the relative concentration of protein expression. The primary antibodies were SYK (AF3314; Affinity Biosciences), p38 (AF4001; Affinity Biosciences), JNK (AF3318; Affinity Biosciences), PI3K (AF3241; Affinity Biosciences), and ERK (AF1015; Affinity Biosciences).

### Data processing and statistical analysis

Statistical analysis was performed using SPSS version 18.0 (SPSS Inc., Chicago, IL, USA) and GraphPad Prism 8.0 (GraphPad Software Inc., La Jolla, CA, USA). Log2 transformed data were used to calculate the difference of proteins using the *t*-test function in the R package (version 3.5.1). The hypergeometric distribution model was used to determine biological pathways that were significantly enriched with DEGs. The Benjamini–Hochberg procedure was used to adjust the p-values to control the false discovery rate (FDR). Fisher exact probability method was used for counting data such as gender, and Wilcoxon-Mann–Whitney U two sample test was used for measuring data such as age. An unpaired *t*-test was used to analyze the differences in RNA content and protein concentration between the LGBLEL and CH groups. P < 0.05 was considered statistically significant.

### Ethics approval and consent to participate

This article does not include patients’ names, pictures, and other personal information. Informed consent was obtained from the patients for publication of this article and any accompanying images.

### Consent for publication

All the authors read and approved the final manuscript for publication.

## Results

### Differential gene expression and GO and KEGG analysis of transcriptome

**Figure 2:**
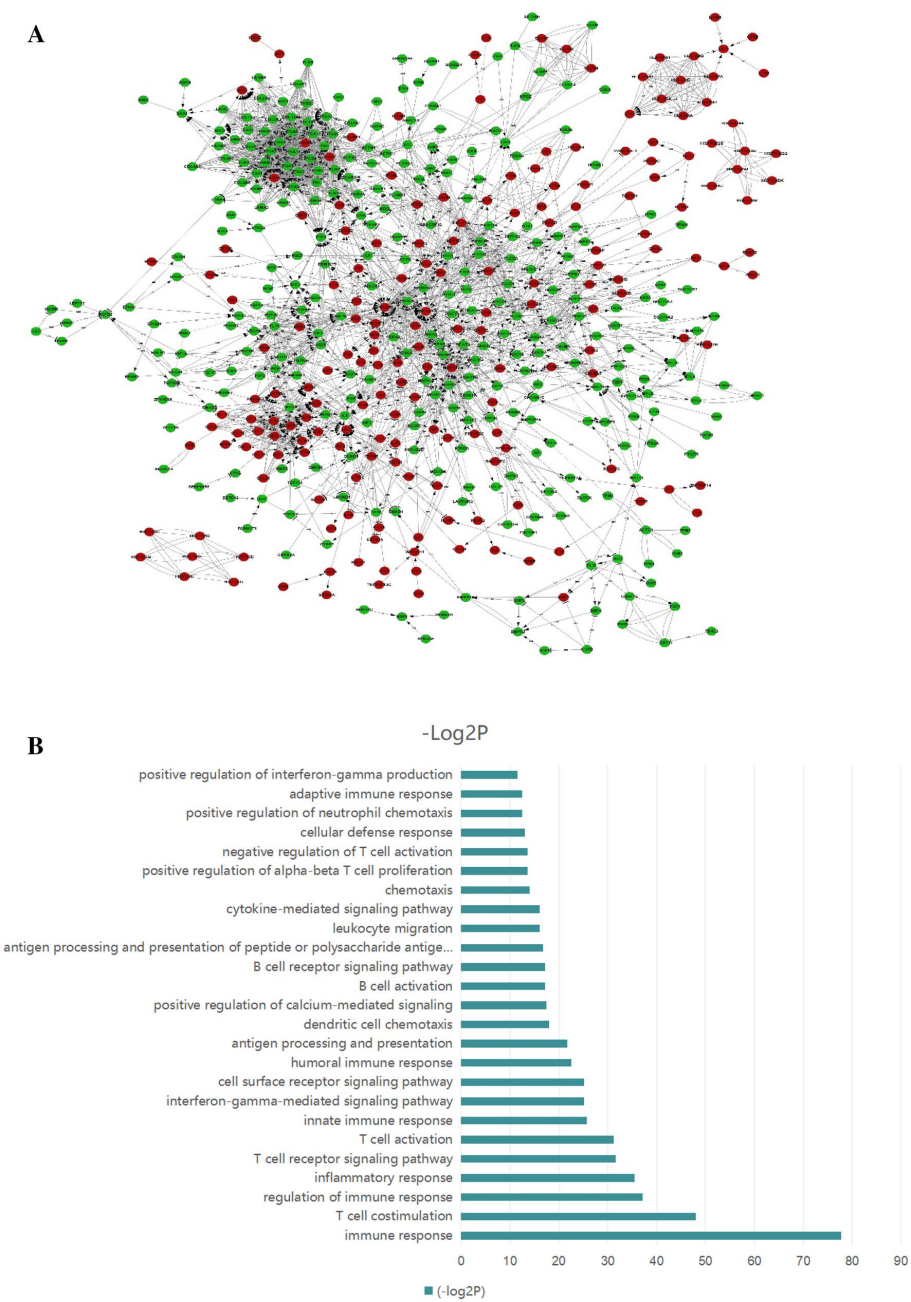

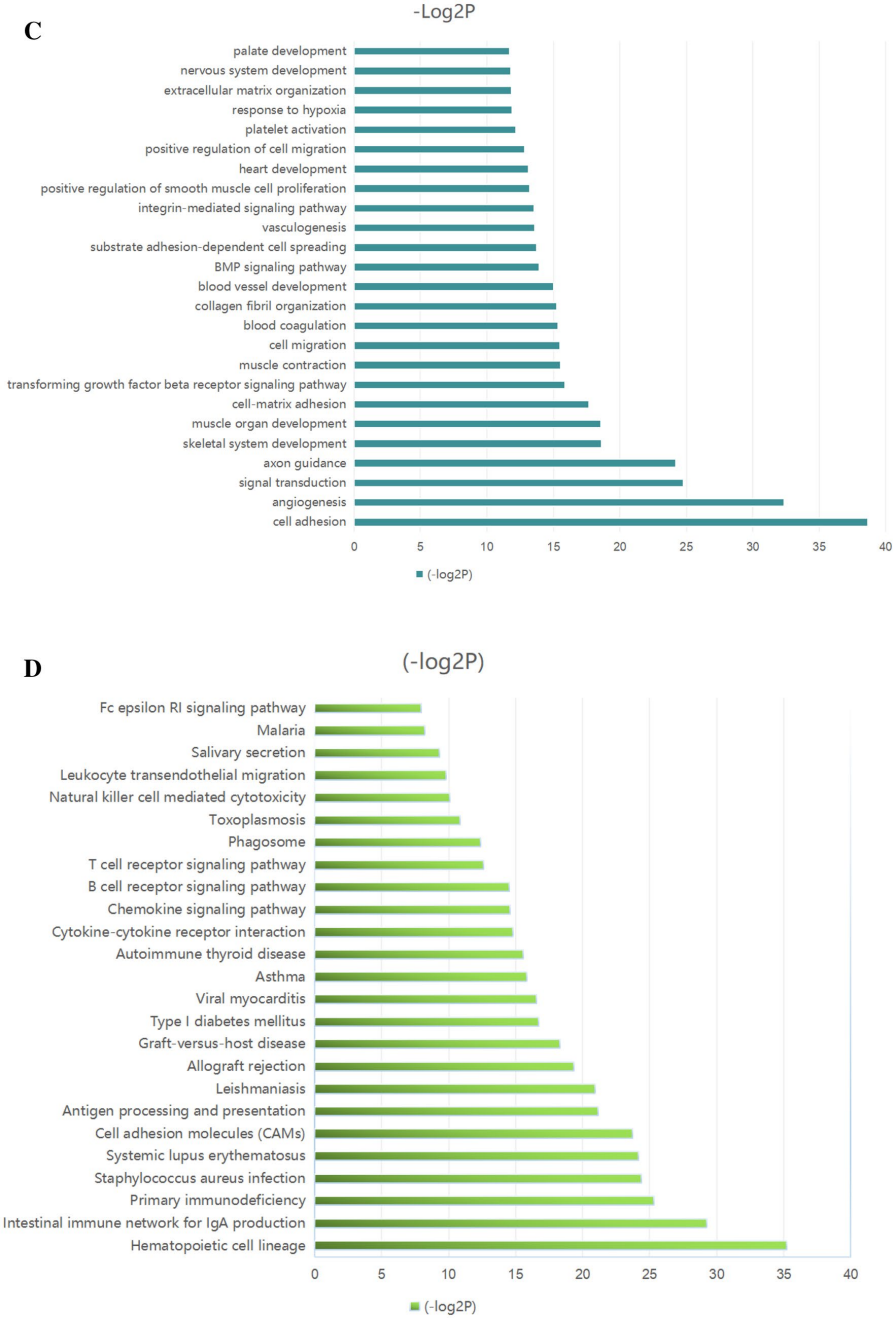

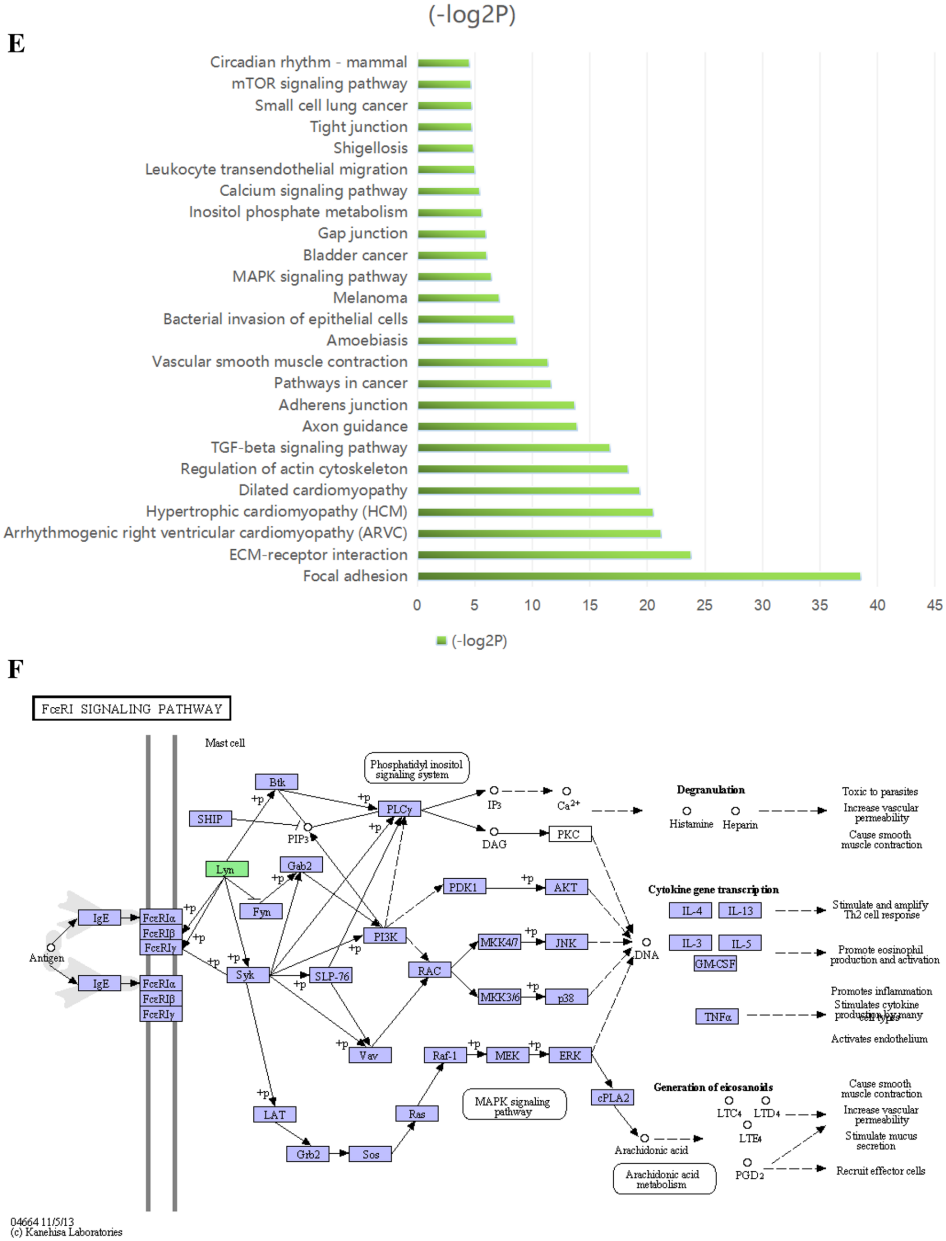
The results of transcriptome analysis. (**A**) Interaction network of DEGs (red: upregulated genes, green: downregulated genes). (**B**) Top 25 enriched biological processes for upregulated DEGs. (**C**) Top 25 enriched biological processes for downregulated DEGs. (**D**) Top 25 pathway enrichment analyses for upregulated DEGs. (**E**) Top 25 pathway enrichment analyses for downregulated DEGs. (**F**) DEGs mapped with KEGG FcεRI signaling pathway (green: upregulated, purple: downregulated).

DEGs between the LGBLEL and CH groups that had |log2FC|> 1 and FDR < 0.01 were selected. There were 5124 differentially expressed genes, among which 3,501 genes were downregulated and 1,623 genes were upregulated. The network interaction analysis for 686 upregulated and 892 downregulated DEGs with confidence scores ≥ 0.9 is shown in Fig. 2A. Compared with the CH group, the downregulated DEGs in the LGBLEL group were primarily related to biological processes associated with development-associated signaling processes, including signal transduction, cell adhesion and differentiation, while the upregulated DEGs were primarily associated with immune signaling functions, including immune response, regulation of immune response, as well as T cell and B cell activation (Fig. 2B,C).

KEGG pathways enrichment for the DEGs was performed using the online functional annotation tool DAVID. The top 25 KEGG pathways that were significantly enriched for the downregulated and upregulated DEGs are shown in Fig. 2D,E. The signaling pathways for upregulated DEGs in the LGBLEL groups were associated with the hematopoietic cell lineage, intestinal immune network for IgA production, primary immunodeficiency, and cell adhesion molecules, while the FcεRI signaling pathway was upregulated in the LGBLEL compared with CH group. The signaling pathways for downregulated DEGs in the LGBLEL group were associated with several processes including focal adhesion, ECM-receptor interaction, and arrhythmogenic right ventricular cardiomyopathy. A map of the DEGs with the KEGG pathways for the FcεRI signaling pathway is exhibited in Fig. 2F.

### SYK, p38, JNK, PI3K, and ERK mRNA expression levels related to the FcεRI signaling pathway were increased in LGBLEL

**Figure 3:**
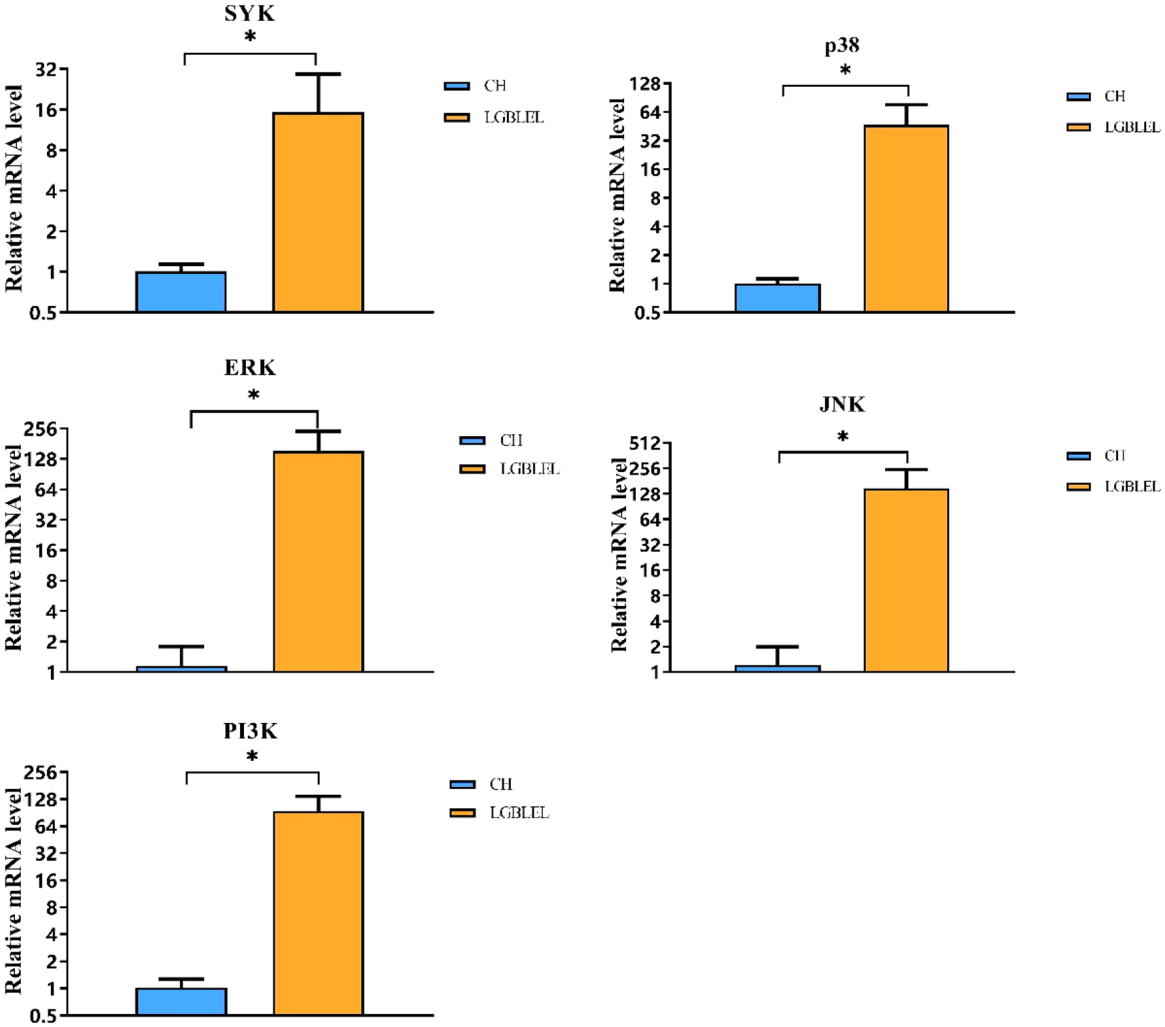
The mRNA expression levels of important genes in the FcεRI signaling pathway. The mRNA expression levels of SYK, P38, JNK, PI3K, and ERK in LGBLEL tissues were significantly increased (P = 0.0066, P = 0.0002, P = 0.0003, P < 0.0001, P < 0.0001) compared with CH tissues. "*" indicates statistical significance.

The previous results indicated that the FcεRI signaling pathway is involved in the pathogenesis of LGBLEL. Therefore, we then wanted to identify and validate the functions of important genes associated with the FcεRI signaling pathway in LGBLEL, including SYK, p38, JNK, PI3K, and ERK. RT-PCR showed that the mRNA expression levels of SYK, P38, JNK, PI3K, and ERK were significantly higher in the LGBLEL compared with the orbital CH group (P = 0.0066; P = 0.0002; P = 0.0003; P < 0.0001; P < 0.0001, respectively) (Fig. 3).

### Immunohistochemical staining showed higher protein expression levels of P-SYK, P-p38, and P-ERK in LGBLEL tissues vs orbital CH tissues

**Figure 4:**
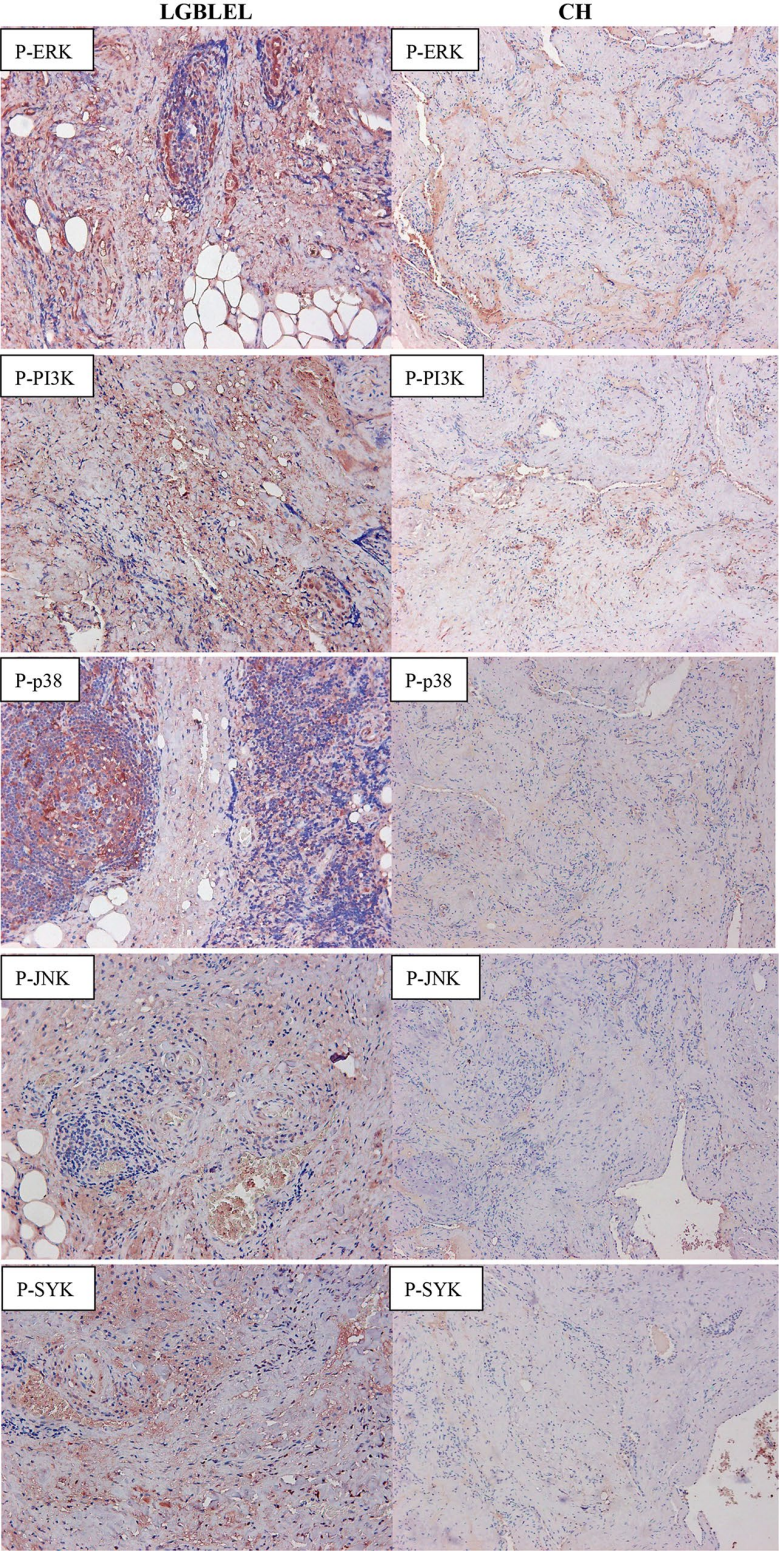
Immunohistochemical staining of proteins related to the FcεRI signaling pathway in LGBLEL and CH. P-Syk, P-p38, and P-ERK proteins are shown as brownish yellow in LGBLEL tissues (SP method, magnification X200), and the expression levels were higher than in orbital CH (SP method, magnification X100). P-JNK and P-PI3K were positively expressed in interstitial tissue, but not in lymphoepithelial lesions in LGBLEL (SP method, magnification X200).

As shown in Fig. 2C, SYK, p38, JNK, PI3K, and ERK play a functional role in the FcεRI signaling pathway via phosphorylation. The results of immunohistochemical staining showed that P-SYK, P-p38, and P-ERK proteins were positively expressed, and the expression levels were significantly higher in LGBLEL tissues than in orbital CH tissues. The positive protein is stained brown and yellow (Fig. 4). However, P-JNK and P-PI3K were positively expressed in LGBLEL interstitial tissue, but not in the lymphoepithelial lesions.

### Western blotting showed higher P-SYK, P-JNK, P-p38, P-PI3K, and P-ERK protein expression in LGBLEL tissues

**Figure 5:**
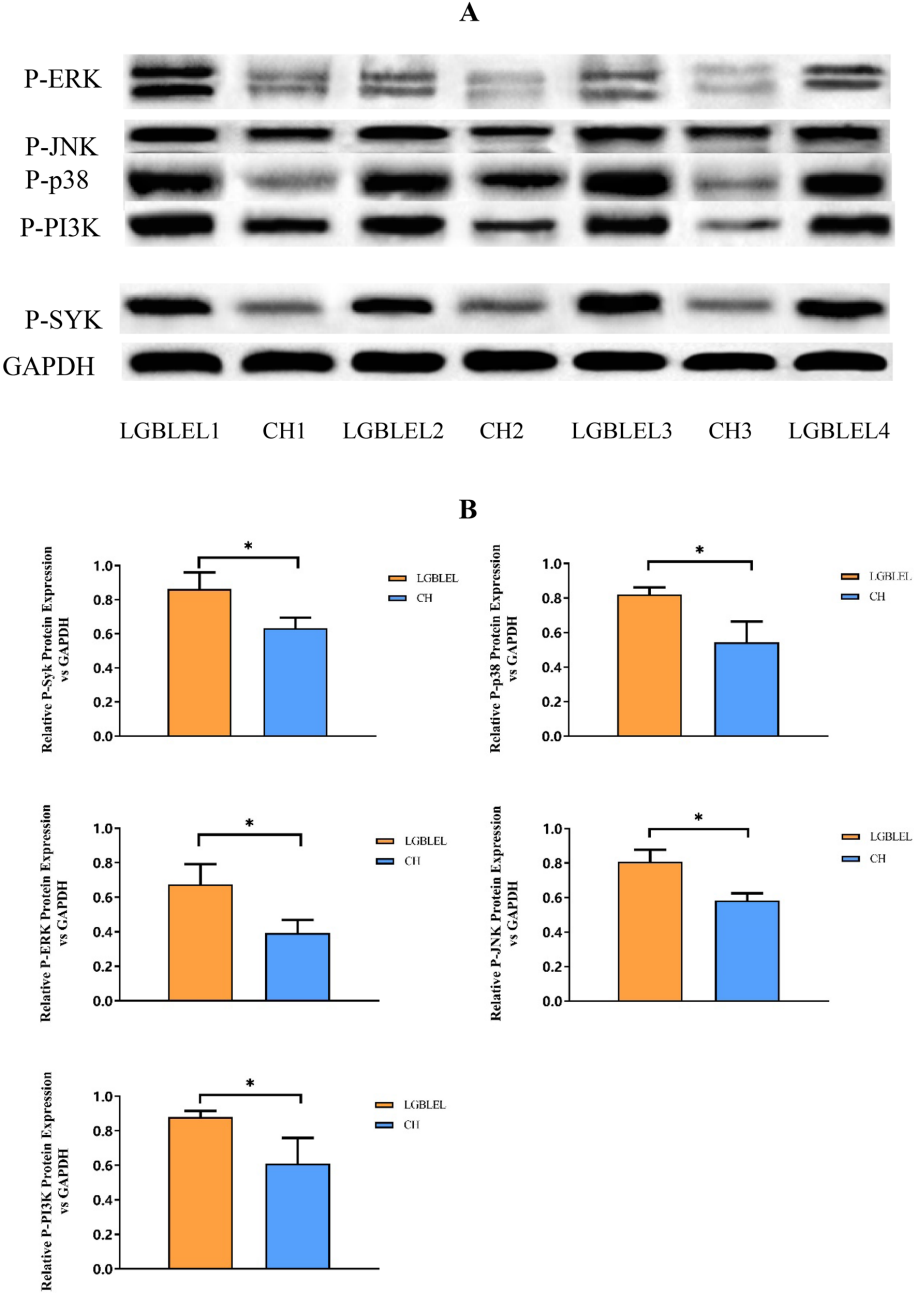
Comparison of protein immunoblotting and protein content between LGBLEL and orbital CH. (**A**) Western blotting showed higher expression of proteins related to the FcεRI signaling pathway in LGBLEL than in orbital CH. (**B**) The protein contents of p-SYK, p-p38, p-JNK, p-PI3K, and p-ERK were significantly higher than in orbital CH (P = 0.0169; P = 0.0074; P = 0.0046; P = 0.0157; P = 0.0156, respectively).

According to the results of western blotting and protein content detection, P-SYK, P-p38, P-JNK, P-PI3K, and P-ERK proteins were highly expressed in LGBLEL tissues and were significantly higher than in the CH group (Fig. 5A). The P-SYK, P-p38, P-JNK, P-PI3K, and P-ERK protein contents in LGBLEL tissues were significantly higher than in CH tissues (P = 0.0169; P = 0.0074; P = 0.0046; P = 0.0157; P = 0.0156, respectively) (Fig. 5B).

## Discussion

The FcεRI signaling pathway has been proven to be associated with allergic responses, inflammatory responses, and autoimmune diseases, such as rheumatoid arthritis and osteoarthritis^21–23^. Allergic diseases such as allergic rhinitis, chronic sinusitis, and asthma are common causes of chronic inflammatory lesions and are associated with the activation of mast cells and basophil granulocytes. LGBLEL is a chronic inflammatory disease, which clinical studies have indicated may be related to diseases including sinusitis and allergic rhinitis. Adzavon et al.^24^ found that the FcεRI signaling pathway may be involved in the mechanism that leads to malignant development in LGBLEL. The occurrence of malignant transformation is one of the pathological processes of LGBLEL; therefore, the FcεRI signaling pathway was examined in this study to further verify its role in LGBLEL pathogenesis.

The transcriptome analysis showed that the FcεRI signaling pathway is involved in the pathogenesis of LGBLEL, and RT-PCR was used to quantitatively analyze the important genes related to the FcεRI signaling pathway. The mRNA expression levels of SYK, p38, JNK, PI3K, and ERK were found to be increased in LGBLEL tissues. Western blotting confirmed that the protein expressions of SYK, p38, JNK, PI3K, and ERK were significantly higher in LGBLEL tissues than in orbital CH tissues. In addition, immunohistochemical staining showed that SYK, p38, and ERK were positively expressed in LGBLEL, though JNK and PI3K were not expressed in the lymphocytes or epithelial cells in BLEL. This study confirms the involvement of the FcεRI signaling pathway in the pathogenesis of LGBLEL.

The FcεRI signaling pathway plays a functional role in various ways. JNK is highly responsive to a diverse array of cellular stimuli, such as inflammatory cytokines, and plays a significant role in innate and adaptive immune responses^25,26^. PI3K can promote pro-inflammatory cytokine production and plays a regulatory role in immune responses^27,28^. Therefore, the negative expression of P-JNK and P-PI3K in immunohistochemical staining may indicate that JNK and PI3K are not the key signaling molecules of the FcεRI signaling pathway but may play an auxiliary role in the mechanism of LGBLEL. SYK is a cytoplasmic tyrosine kinase that is involved in many cellular signaling processes and drives immune inflammation^29^. Studies have shown that SYK, JNK, and ERK play important regulatory roles in B cell receptor signaling pathways^30^. SYK, PI3K, JNK, and p38 can jointly affect T cell activation and participate in the T cell receptor signaling pathway^31–33^. SYK and JNK can participate in complement system-mediated phagocytosis, T cell activation, and inflammatory responses^34,35^. Previous studies have confirmed that the B cell receptor signaling pathway, the T cell receptor signaling pathway, and the complement signaling pathway are involved in LGBLEL pathogenesis^1,11,12^. The B cell receptor, T cell receptor, and FC receptor are classical immune-related receptors^29^. Therefore, we hypothesized that these signaling pathways may interact with FcεRI-mediated signaling pathways to promote inflammatory infiltration of lacrimal tissue, leading to lacrimal gland enlargement and fibrosis.

IgG4-RD is considered to be a Th2 cell-dominated disease, and cytokines, such as IL-4 and IL-13, can promote IgG4 conversion^36^. Studies of IgG4-RD have shown infiltration of many CD4 + and CD25 + Treg cells in the affected tissues and an increase in the number of CD4 + and CD25 + Treg cells in the blood, suggesting activation of Treg cells in IgG4-RD^37^. Treg cells and Th2 cells can produce inflammatory mediators, such as IL-10, IL-4, IL-5, IL-13, and transforming growth factor-β, leading to chronic inflammation. IgE-mediated activation of the FcεRI signaling pathway can directly lead to increased secretion of cytokines, such as IL-3, IL-4, IL-5, IL-13, and tumor necrosis factor-α, leading to Th2 cell activation, eosinophilic activation, and aggravation of the inflammatory response^14,15^. The release of inflammatory mediators can activate the B cell receptor pathway, T cell receptor pathway, and complement signaling pathway, leading to lacrimal gland injury, tumor-like hyperplasia, and dysfunction.

In this study, we demonstrated the role of IgE-mediated FcεRI signaling in the pathogenesis of LGBLEL. Studies have shown that, in the IgG-mediated replacement pathway, IgG acts on a variety of immune cells by cross-linking with FcγR^16^. Beutier et al. showed that FcγRIII receptors were the main receptors for IgG-dependent passive systemic allergic reactions induced by IgG1, IgG2a, and IgG2b antibodies, and that macrophages, mast cells, basophils, and neutrophils participated in the IgG-mediated pathway through the recruitment of FcγR^38^. IgG immune complex can also cause the release of complement C3a, C5a, and C5b-9, which then activates mast cells and basophils, causing cell degranulation and release of soluble mediators^39^. The elevated expression of IgG and IgG-subtypes in the serum of some LGBLEL patients suggests that the IgG-mediated replacement pathway and complement system may also play important roles in the pathogenesis of LGBLEL.

Due to the difficulty in obtaining normal lacrimal gland tissue, this study used orbital cavernous hemangioma as the control group, which is consistent with previous studies^11,12^. The role of the IgG-mediated substitution pathway and complement system in the pathogenesis of LGBLEL and the relationship between the FcεRI signaling pathway and other signaling pathways need to be further studied and verified. The immunohistochemical staining results of JNK and PI3K may have included false negatives due to the influence of antibodies, tissue locations, and experimental operations. In addition, the expression levels of inflammatory mediators still need to be determined in serum from LGBLEL patients. The pathogenesis of LGBLEL is complex and involves multiple signaling pathways. Based on previous studies, the results of this study indicate that the FcεRI signaling pathway participates in LGBLEL pathogenesis and provides new evidence for understanding the pathogenesis of LGBLEL.

## Supplementary Information

Supplementary Information.

## Data Availability

All files are available from the GEO database (GSE76497).

## Abbreviations

LGBLEL: Lacrimal gland benign lymphoepithelial lesions
FcεRI: FcepsilonRI
CH: Cavernous hemangioma
IL: Interleukin
Th2: Helper T2
FDR: False discovery rate
DEGs: Differentially expressed genes
GO: Gene ontology
KEGG: Kyoto encyclopedia of genes and genomes
DAVID: Database for Annotation, Visualization and Integrated Discovery
IgG4-ROD: IgG4-related ocular disease
RT-PCR: Reverse transcription polymerase chain reaction
SYK: Spleen tyrosine kinase
p38: Mitogen-activated protein kinase, MAPK
JNK: C-Jun N-terminal kinase
PI3K: Phosphoinositide 3-kinase
ERK: Extracellular signal-regulated kinases
