# Supplementary figures and images for: The FcεRI signaling pathway is involved in the pathogenesis of lacrimal gland benign lymphoepithelial lesions as shown by transcriptomic analysis
Source: Sci Rep. 2021 Nov 8;11:21853. doi: 10.1038/s41598-021-01395-z (PMC8576038; doi:10.1038/s41598-021-01395-z)

P-ERK
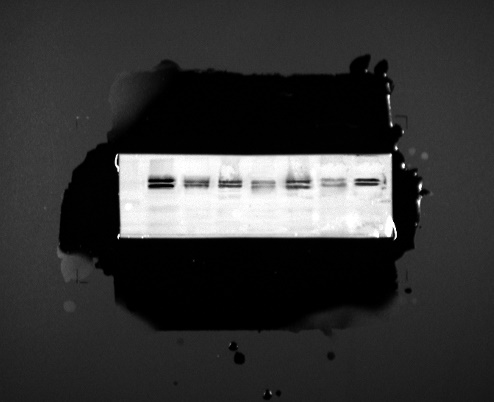


P-JNK
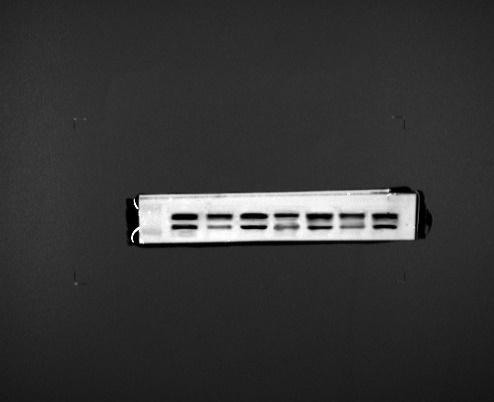


P-p38
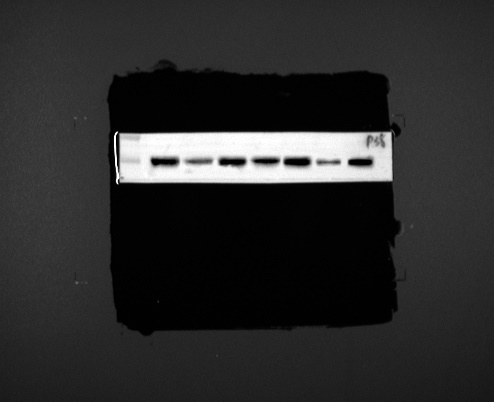


P-PI3K
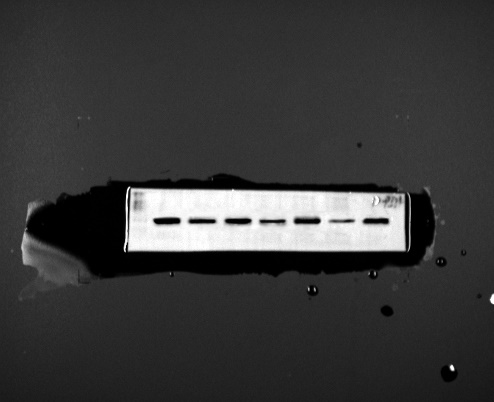


P-SYK
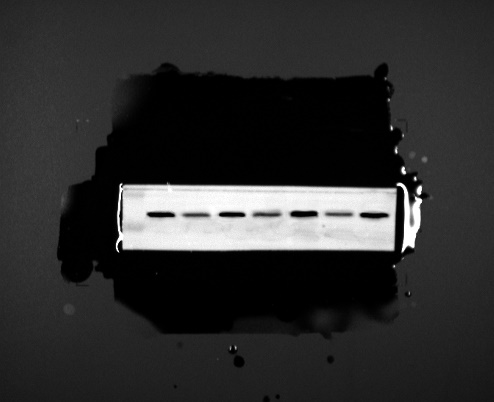


GAPDH
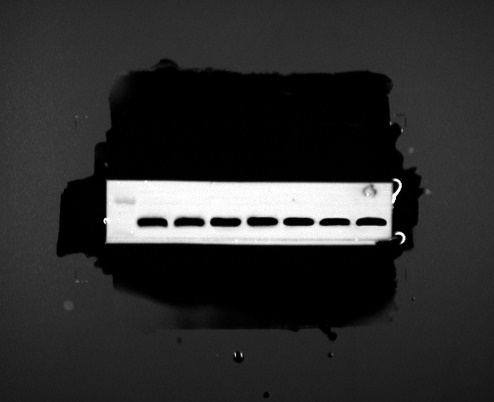

Supplement: Supplementary file 1 — Supplementary Information. [file 41598_2021_1395_MOESM1_ESM.doc]
